# RNA-Seq reveals novel genes and pathways involved in bovine mammary involution during the dry period and under environmental heat stress

**DOI:** 10.1038/s41598-018-29420-8

**Published:** 2018-07-23

**Authors:** Bethany Dado-Senn, Amy L. Skibiel, Thiago F. Fabris, Y. Zhang, Geoffrey E. Dahl, Francisco Peñagaricano, Jimena Laporta

**Affiliations:** 1 Department of Animal Sciences, University of Florida, Gainesville, FL USA; 2 Interdisciplinary Center for Biotechnology Research, University of Florida, Gainesville, FL USA; 3 University of Florida Genetics Institute, University of Florida, Gainesville, FL USA

## Abstract

The bovine dry period is a dynamic non-lactating phase where the mammary gland undergoes extensive cellular turnover. Utilizing RNA sequencing, we characterized novel genes and pathways involved in this process and determined the impact of dry period heat stress. Mammary tissue was collected before and during the dry period (−3, 3, 7, 14, and 25 days relative to dry-off [day 0]) from heat-stressed (**HT**, n = 6) or cooled (**CL**, n = 6) late-gestation Holstein cows. We identified 3,315 differentially expressed genes (**DEGs**) between late lactation and early involution, and 880 DEGs later in the involution process. DEGs, pathways, and upstream regulators during early involution support the downregulation of functions such as *anabolism* and *milk component synthesis*, and upregulation of *cell death*, *cytoskeleton degradation*, and *immune response*. The impact of environmental heat stress was less significant, yet genes, pathways, and upstream regulators involved in processes such as ductal branching morphogenesis, cell death, immune function, and protection against tissue stress were identified. Our research advances understanding of the mammary gland transcriptome during the dry period, and under heat stress insult. Individual genes, pathways, and upstream regulators highlighted in this study point towards potential targets for dry period manipulation and mitigation of the negative consequences of heat stress on mammary function.

## Introduction

In dairy cows, the dry period is a six to eight-week non-lactating state initiated between lactations that allows for optimal milk yield in the subsequent lactation through the turnover of worn, senescent mammary epithelial cells (**MEC**) with new, active cells^1^. It consists of three phases known as active involution, steady state involution, and redevelopment. Involution is the natural process whereby the mammary gland transitions from a lactating to a non-lactating state^2^. It begins after the cessation of milk removal and is characterized by a decrease in milk secretion and rise in mammary pressure, apoptosis and autophagy of MEC, and immune response^3–5^. Involution continues for approximately 21 d, followed by redevelopment of the mammary gland until calving^6^.

The onset of involution triggers the expression of genes and pathways that function to increase cell death and immune signals. Downregulated pathways during involution include prolactin signaling (via the inactivation of *STAT5*, a cell proliferation and differentiation regulator^7,8^) and insulin-like growth factor (**IGF;** via the upregulation of IGF-binding protein (**IGFBP**)5, a regulator of cell apoptosis and tissue remodeling^9^). The redevelopment phase is a mammogenic period where upregulation of genes, such as *IGF1* and *IGFBP3*, promote cell proliferation and turnover to increase MEC number and secretory capacity in preparation for colostrogenesis and lactation^1,5^. Key candidate genes have been well characterized in rodent models. In dairy cattle, limited studies have utilized microarrays and qRT-PCR to evaluate the molecular events occurring in the mammary gland during a typical dry period of pregnant cows^5^, during forced involution of non-pregnant cows at peak lactation^10,12^, and during gradual involution of non-pregnant cows at peak lactation^11^. These studies report an overall upregulation of genes related to cell turnover, oxidative stress, tissue remodeling, and inflammation and downregulation of cell survival signaling and biosynthesis of milk constituents during involution and upregulation of cellular proliferation later during redevelopment. However, a more thorough characterization of the entire bovine mammary transcriptome through *in vivo* models is lacking.

Perturbations, such as impaired nutrition and poor management, during the dry period may alter the involution process and affect cow performance. Exposure of dairy cows to environmental heat stress during the dry period decreases milk production in the subsequent lactation^13,14^. This phenomenon has been partially attributed to reduced autophagy in the early dry period^15^, decreased cell proliferation in the late dry period^14^, and altered alveolar microstructure^16^. Bovine MEC exposed to acute heat stress *in vitro* downregulate genes related to cell cycle, focal adhesion and cytoskeleton activity, cell biosynthesis and metabolism, ductal branching, and morphogenesis and upregulate genes involved in stress response and protein repair^17,18^. Whereas the effect of heat stress on cellular processes and *in vitro* gene expression has been studied, its impact on the mammary gland transcriptome through *in vivo* models has yet to be elucidated for the bovine.

The aim of this study was to discover and characterize novel genes, pathways, and upstream regulators involved in mammary gland involution and redevelopment during the dry period and to determine how heat stress affects this dynamic process in the dairy cow by utilizing RNA-Sequencing. We hypothesize that, relative to cooled cows, cows exposed to environmental heat stress will experience alterations in expression of key genes and pathways required for normal involution and redevelopment, compromising mammary function and milk production in the subsequent lactation.

## Results

### Physiological parameters and milk yield

Physiological parameters and production data of the cows used in this study are reported in Fabris *et al*.^19^. Briefly, heat-stressed (**HT**) and cooled (**CL**) pens had similar temperature humidity index (**THI**) which was never lower than 68 at any time during the experimental period. Cows provided with active cooling during the dry period had a tendency (*p* ≤ 0.10) toward higher feed intake (11.0 vs. 10.3 ± 0.46 kg/d, *p* = 0.10; CL vs. HT respectively), had lower rectal temperature (38.92 vs. 39.31 ± 0.05 °C, *p* < 0.01), and had reduced respiration rates (45.2 vs. 77.2 ± 1.59 breaths/min, *p* < 0.01) compared with heat stressed cows. Thus, heat stress was effective in inducing physiological changes. On average, cows provided with active cooling during the dry period tended to have increased milk production, yielding 4.8 kg more milk over 9 weeks compared to heat stressed cows (40.7 vs 35.9 ± 1.6 kg/d, *p* = 0.09).

### Mapping statistic summary

RNA-Sequencing (**RNA-Seq**) technology was used to analyze genome-wide gene expression of mammary samples collected on day (**D**) −3, 3, 7, 14, and 25 relative to dry-off (D0) for cows under HT or CL conditions. Multidimensional scaling plots show the relative similarities of the samples (Supplementary Fig. S1). Through Illumina sequencing we acquired roughly 34 million single-ended reads per sample. Approximately 81% of the reads were successfully mapped to the bovine genome. Among these aligned reads, 98% were mapped to unique genomic regions. Only uniquely mapped reads were considered in the analysis. Sequencing data can be accessed through NCBI GEO with accession number GSE108840.

### Differentially expressed genes and pathways across the dry period

**Figure 1:**
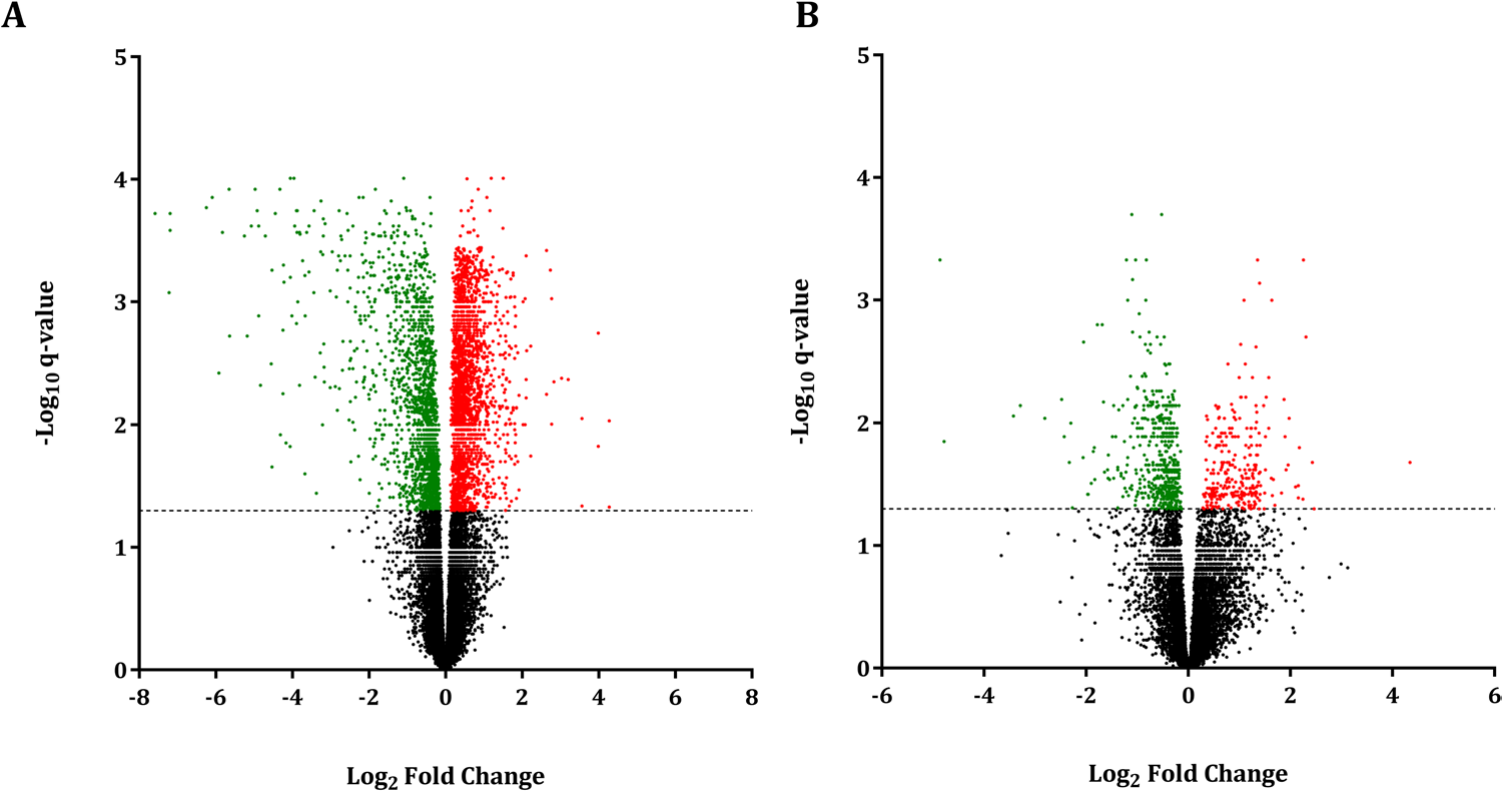
Volcano plot of differentially expressed genes in bovine mammary tissue during early involution. Differential gene expression in the bovine mammary gland contrasting (**A**) D3 vs. D-3 (n = 12, early involution vs. late lactation) and (**B**) D7 vs. D3 (n = 12, first week of involution). D0 indicates dry-off (~46 d relative to expected calving). Cut-off criteria for DEG significance was FDR ≤ 5%. The y-axis displays the -log_10_ q-value for each gene, while the x-axis displays the log_2_ fold change for that gene relative to D3 (**A**) or D7 (**B**). Red dots indicate upregulation, green dots indicate downregulation, and black dots indicate non-significance relative to (**A**) D3 or (**B**) D7.

**Figure 2:**
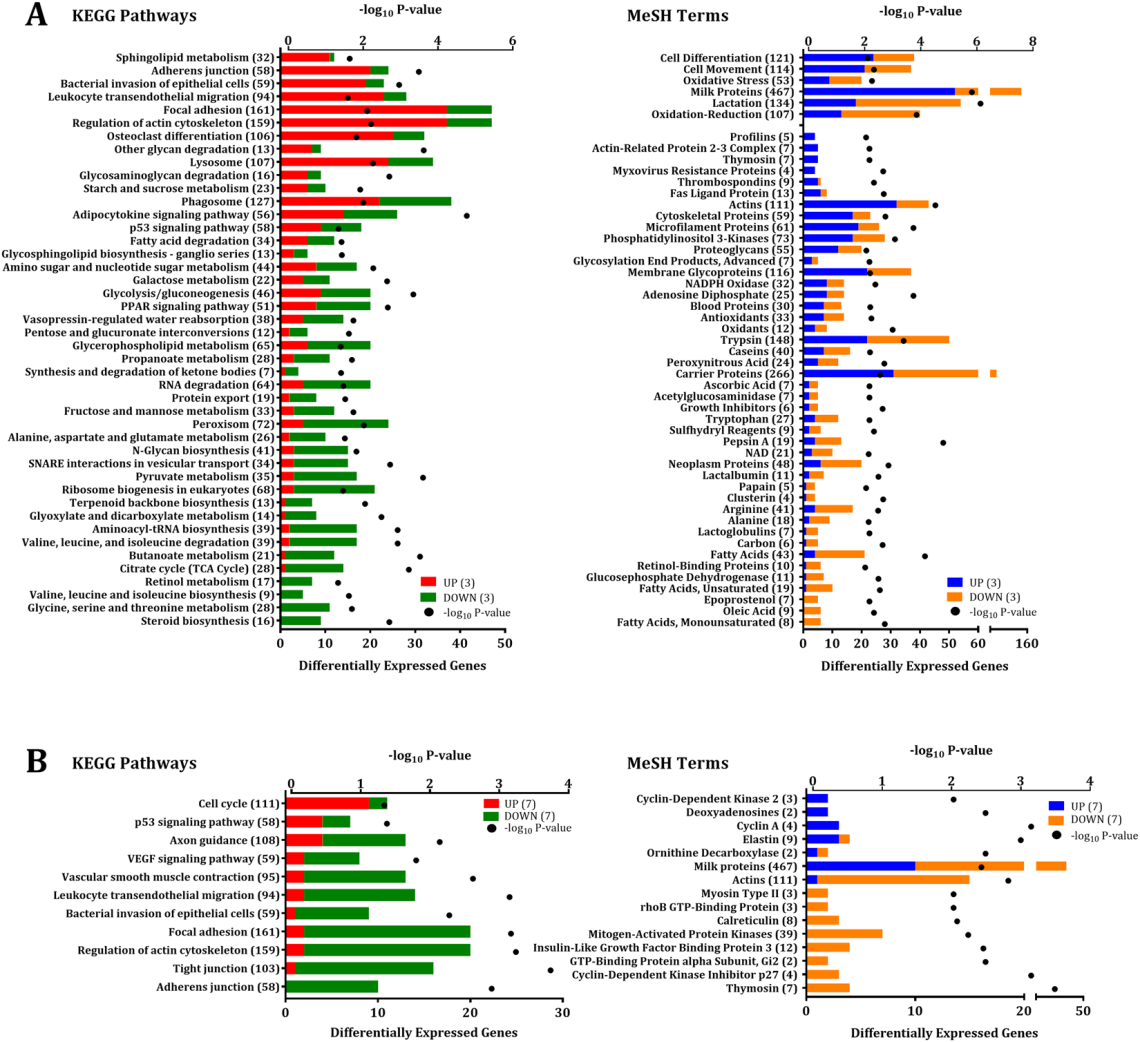
Significantly enriched Kyoto Encyclopedia of Genes and Genomes (KEGG) pathways and Medical Subject Headings (MeSH) terms in bovine mammary tissue during early involution. Enriched KEGG pathways and MeSH terms among differentially expressed genes (DEG) in the bovine mammary gland contrasting (**A**) D3 vs. D-3 (n = 12, early involution vs. late lactation) and (**B**) D7 vs. D3 (n = 12, first week of involution). D0 indicates dry-off (~46 d relative to expecting calving). DEG significance was set at FDR ≤ 5%, and pathway/term significance was set at *p* ≤ 0.01 (Fisher’s exact test). The y-axis displays the names and the total number of genes of each pathway/term. The x-axis displays the total significance of enrichment (–log_10_ p-value) and the number of DEG within each pathway/term with expression at (**A**) D3 relative to D-3 or (**B**) D7 relative to D3. Red and blue bars indicate proportion of upregulated DEG while green and orange bars indicate proportion of downregulated DEG.

The main effect of time relative to dry-off on the mammary gland transcriptome was analyzed, comparing D3 vs. D-3, D7 vs. D3, D14 vs. D7, and D25 vs. D14. When comparing D3 (initiation of involution) vs. D-3 (late lactation) 3,315 genes were differentially expressed (**DEGs**), of which 1,311 were upregulated, and 2,004 were downregulated at D3 relative to D-3 (false-discovery rate (**FDR**) ≤ 5%, Fig. 1A, Supplementary Table S1). These DEGs were associated with 44 Kyoto Encyclopedia of Genes and Genomes (**KEGG**) pathways and 51 Medical Subject Heading (**MeSH**) terms (*p* ≤ 0.01, Fig. 2A, Supplementary Table S2). KEGG pathways with a high percentage of DEGs upregulated at D3 were related to cytoskeleton and cellular degradation and immune response, while pathways with a greater ratio of downregulated DEGs were associated with anabolism and amino acid biosynthesis and metabolism. Similarly, MeSH terms related to cytoskeletal proteins and cellular differentiation and movement had a high proportion of DEGs upregulated at D3, while terms with a greater number of downregulated DEGs at D3 were associated with lactation, milk proteins, and amino acids.

There were fewer DEGs when comparing D7 vs. D3, which captures the first week of involution. We identified 880 DEGs between these time points, 292 of which were upregulated and 588 of which were downregulated at D7 relative to D3 (FDR ≤ 5%, Fig. 1B; Supplementary Table S3). These DEGs were grouped into 11 enriched KEGG pathways and 14 MeSH terms (*p* ≤ 0.01, Fig. 2B; Supplementary Table S4). Only one KEGG pathway, cell cycle, had a high proportion of DEGs that were upregulated at D7. The other ten pathways had a greater ratio of DEGs that were downregulated, and these were associated with cytoskeleton degradation and immunity. DEGs in MeSH terms related to cyclin were exclusively upregulated at D7, while the majority of DEGs in MeSH terms such as actin and kinases were downregulated at D7. Interestingly, the majority of KEGG pathways and MeSH terms had a higher percentage of downregulated DEGs at D7 compared to D3, and 6 out of these 11 KEGG pathways were simultaneously enriched in the D3 vs. D-3 comparison (e.g. *regulation of actin cytoskeleton*, *focal adhesion*, *adherens junction*, *p53 signaling pathway*, *bacterial invasion of epithelial cells*, and *leukocyte transendothelial migration*) indicating a common pattern of regulation during the first week of involution.

As involution progressed to steady state and D14 vs. D7 was compared, there were no DEGs at a FDR ≤ 5%. Similarly, when comparing D25 to D14 to capture the mammary gland redevelopment phase, there were no DEGs at a FDR ≤ 5%. A complete list with all genes for these time point comparisons can be found in Supplementary Tables S5 and S6.

### Ingenuity Pathways Analysis (IPA) upstream regulator and gene network analysis

**Figure 3:**
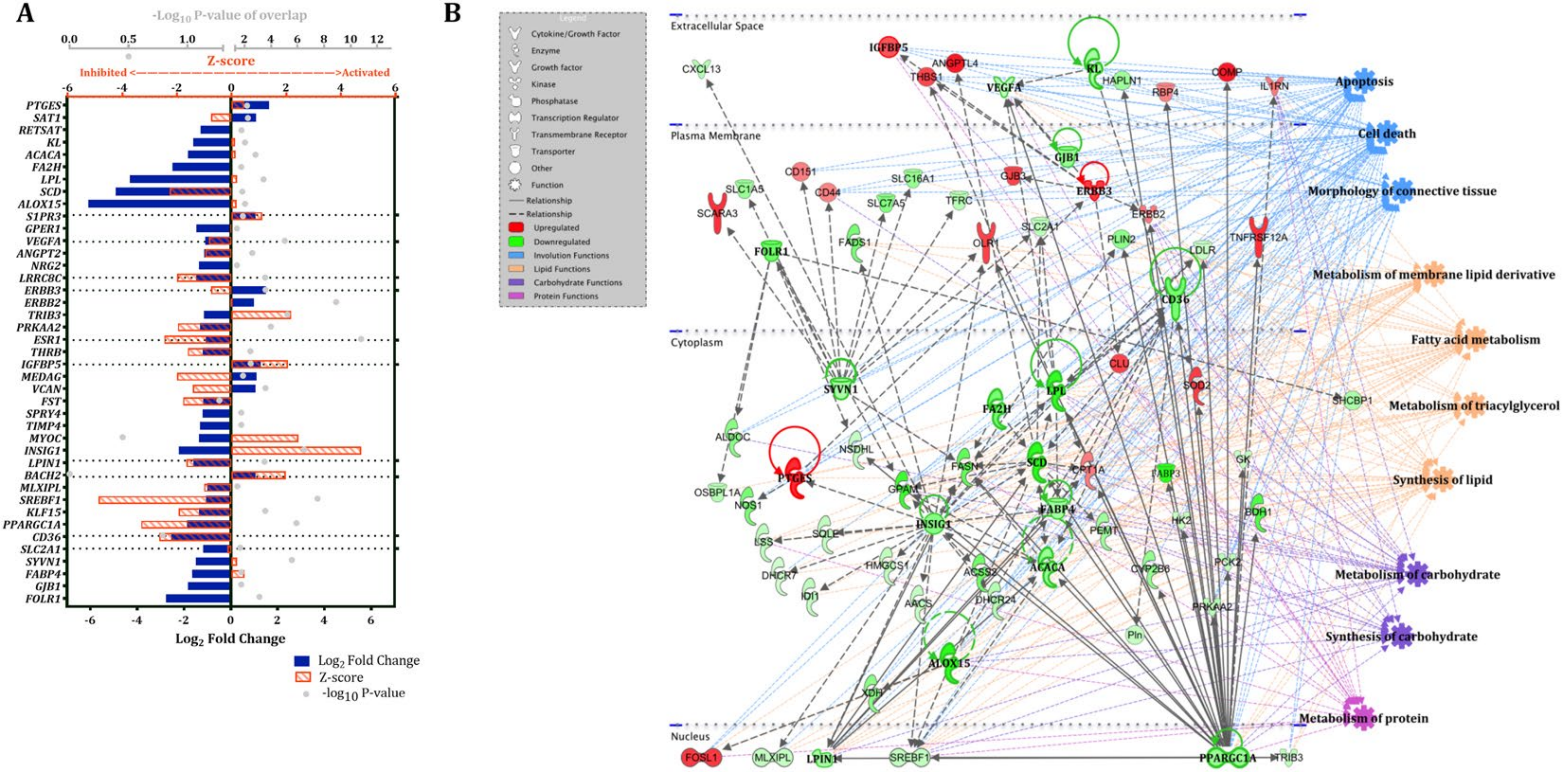
Ingenuity Pathway Analysis (IPA) upstream regulators and summary network in bovine mammary tissue comparing D3 vs. D-3 relative to dry-off. Significant upstream regulators and network in the bovine mammary gland contrasting early involution vs. late lactation (D3 vs. D-3, n = 12). D0 indicates dry-off (~46 d relative to expecting calving). IPA predicts causal effects among upstream regulators and the downstream targets, both of which are differentially expressed genes (DEGs) within the dataset. The DEG significance was set at FDR ≤ 5%, and the upstream regulator significance of enrichment at *p* ≤ 0.05 with log_2_ fold change ≥ |1.0|. (**A**) Upstream regulators are grouped by functional categories with log_2_ fold change (equivalent to expression log ratio) in blue bars, Z-score (activated: > 2, inhibited: < −2) in orange bars, and significance of enrichment (–log_10_ P-value) in gray dots. (**B**) The summary network depicts the interactions between upstream regulators, downstream genes, and physiological functions. Red and green molecules indicate upregulated and downregulated genes at D3, respectively, relative to D-3. Figure legend displays molecules and function symbol types and colors. The functional networks were generated through IPA (QIAGEN Inc., https://www.qiagenbio-informatics.com/products/ingenuity-pathway-analysis)^58^.

Upstream regulators and summary networks for D3 vs. D-3 and D7 vs. D3 were generated utilizing IPA (QIAGEN Inc., https://www.qiagenbioinformatics.com/products/ingenuitypathway-analysis). The list of 2,816 mapped DEGs for D3 vs. D-3 generated a catalog of 179 predicted biological upstream regulators through IPA. After restricting the analysis to those differentially expressed within our dataset with log_2_ fold change ≥ |1.0|, 41 significant upstream regulators were revealed (Fig. 3A). The network analysis of upstream regulators and corresponding downstream genes relative to D3 revealed the participation in functions related to involution and metabolism of lipids, carbohydrates, and proteins (Fig. 3B).

**Figure 4:**
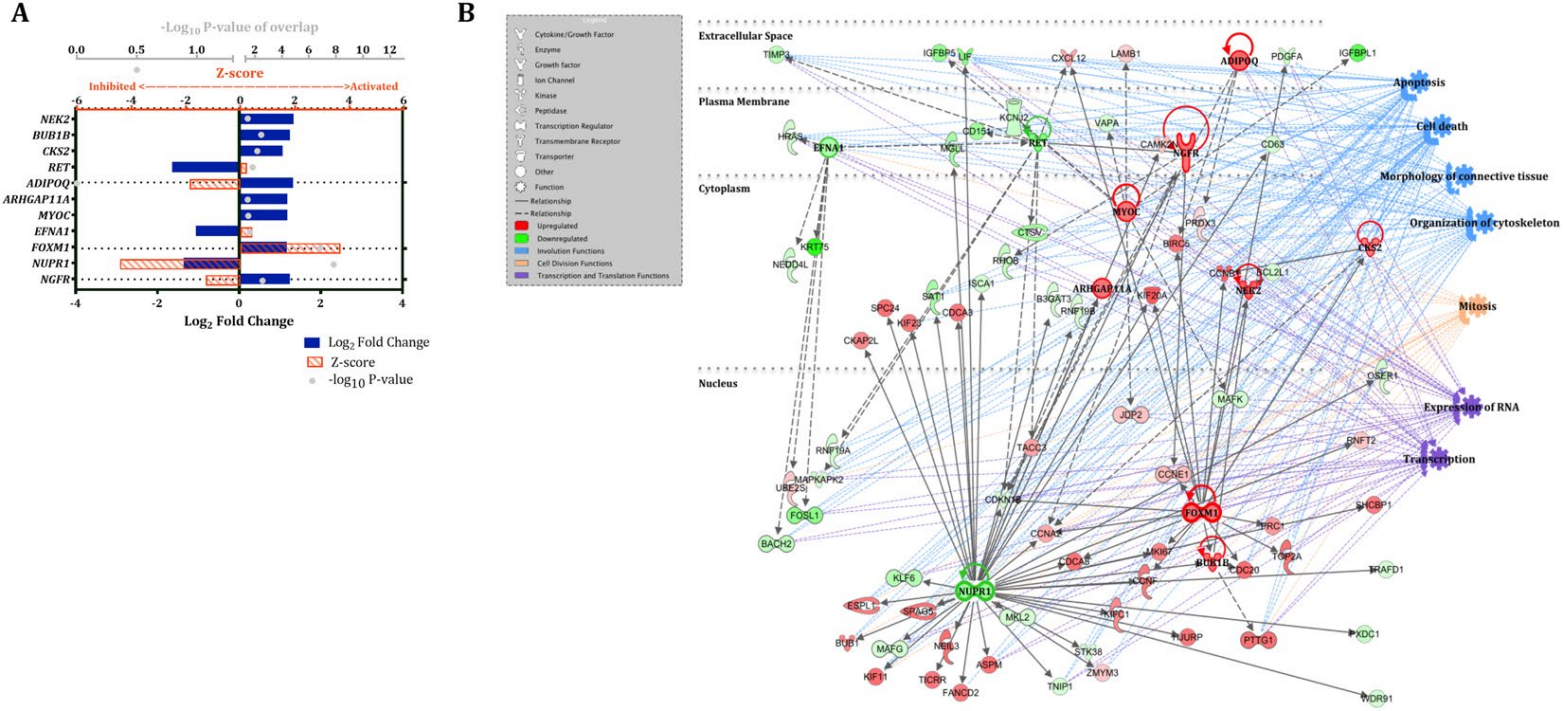
Ingenuity Pathway Analysis (IPA) upstream regulators and summary network in bovine mammary tissue comparing D7 vs. D3 relative to dry-off. Significant upstream regulators and network in the bovine mammary gland during the first week of involution (D7 vs. D3, n = 12). D0 indicates dry-off (~46 d relative to expecting calving). The DEG significance was set at FDR ≤ 5%, and upstream regulator significance of enrichment at p ≤ 0.05 with log2 fold change ≥ |1.0|. (**A**) Upstream regulators are grouped by functional categories with log2 fold change (equivalent to expression log ratio) in blue bars, Z-score (activated: > 2, inhibited: < −2) in orange bars, and significance of enrichment (–log10 P-value) in gray dots. (**B**) The summary network depicts the interactions between upstream regulators, downstream genes, and physiological functions. Red and green molecules indicate upregulated and downregulated genes at D7, respectively, relative to D3. Figure legend displays molecules and function symbol types and colors. The functional networks were generated through IPA (QIAGEN Inc., https://www.qiagenbio-informatics.com/products/ingenuity-pathway-analysis)^58^.

As involution progressed (D7 vs. D3 comparison), there were fewer upstream regulators expressed. From 748 mapped DEGs, a list of 556 predicted biological upstream regulators was obtained through IPA. After restricting the analysis to those differentially expressed within our dataset with log_2_ fold change ≥ |1.0|, 11 were significantly different and the majority was upregulated at D7 (Fig. 4A). The network analysis of these 11 upstream regulators and corresponding downstream genes relative to D7 indicates that these regulators play a role in involution, cell division, and transcription and translation (Fig. 4B).

### Differentially expressed genes and upstream regulators impacted by heat stress

**Figure 5:**
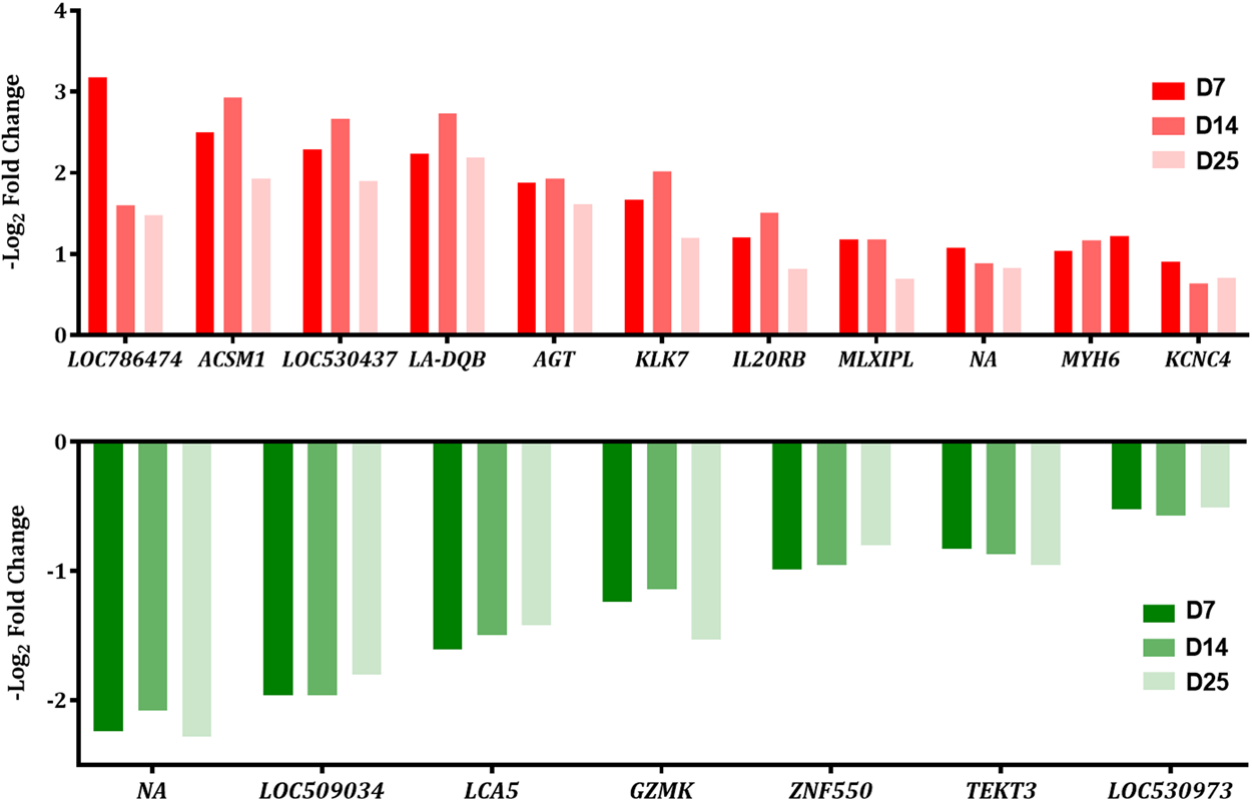
Characterization of differentially expressed genes in bovine mammary tissue between heat-stressed (HT) and cooled (CL) dairy cattle across the dry period. Differentially expressed genes (DEGs, nominal *p* ≤ 0.005, absolute log fold change ≥ 0.5) in the bovine mammary between heat-stressed (n = 6) and cooled (n = 6) dairy cattle consistently up- or downregulated at D7, 14, and 25 relative to dry-off (D0, ~46 d relative to expecting calving). Expression is quantified in HT relative to CL cows. Red indicates upregulation, green indicates downregulation. The y-axis displays the -log_2_ fold change of each DEG and the x-axis lists the gene name.

Differentially expressed genes between dry period HT and CL cows at each specific time point (e.g. D3, 7, 14, and 25 days relative to dry-off) were evaluated. When using a FDR ≤ 5%, the only significant DEG was a non-annotated gene at D25 (log_2_FC = −3.95 and *q* < 0.0001). Using UCSC Genome Browser and NCBI, we identified this non-annotated gene as a long non-coding RNA (**lncRNA**) at position chr7: 61592484–61595879. The Sequence-Structure Motif Base Pre-miRNA Prediction Webserver was used to discern pre-microRNAs (**miRNA**), corresponding mature miRNA seed regions, and the miRNA secondary structures within the lncRNA sequence^20,21^. The program utilizes PriMir filtration and Mirident software to screen and confirm candidate pre-miRNA sequences by score matrix based on features in sequence or structure of known pre-miRNAs. The program revealed 7 mature miRNA seed regions and their secondary structures. According to the bioinformatics program TargetScan utilizing the human database^22^, these seed regions regulate 1,159 downstream target genes (Supplementary Table S7). To explore subtler biological changes due to environmental heat stress, a less stringent approach identified 180 DEGs when comparing HT to CL (9, 115, 27 and 29 DEGs at D3, 7, 14 and 25, respectively; *p* ≤ 0.005 and log_2_ fold change ≥ |0.5|; Supplementary Table S8). From D7 to D25, 11 genes were consistently upregulated and 7 consistently downregulated in HT cows relative to CL (Fig. 5) and related to functions such as ductal branching morphogenesis, inflammatory response, and cell death. Upstream regulators and their resultant networks for HT vs. CL cows at D7 were determined using IPA (QIAGEN Inc., https://www.qiagenbioinformatics.com/products/ingenuitypathway-analysis), and can be found in Supplementary Figure S2.

### Validation of RNA-Seq results with quantitative real-time PCR (qRT-PCR)

**Figure 6:**
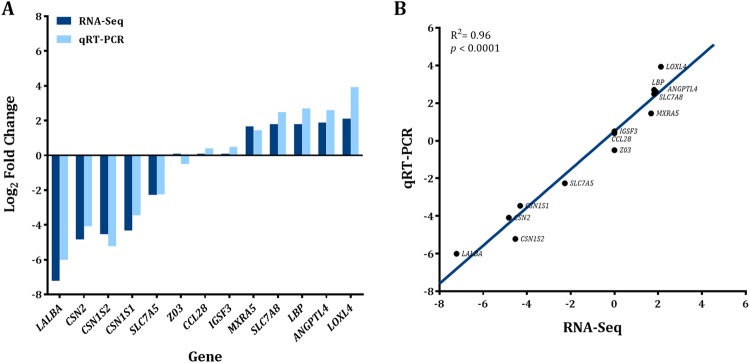
Validation of RNA-Sequencing results by quantitative RT-PCR. (**A**) Log_2_ fold change comparison of RNA-Seq (dark blue bars) and quantitative real-time PCR (qRT-PCR, light blue bars) for five differentially expressed genes downregulated at D3 (*LABLA*, *CSN2*, *CSN1S2*, *CSN1S1*, *SLC7A5*; n = 12), five genes upregulated at D3 (*MXRA5*, *SLC7A8*, *LBP*, *ANGPTL4*, *LOXL4*; n = 12), and 3 genes with similar expression (*CCL28*, *IGSF3*, *ZO;3*, n = 12) when comparing D3 vs. D-3 relative to dry-off (D0, ~46 d relative to calving). (**B**) Correlation between RNA-Seq and RT-PCR gene expression (R^2^ = 0.96, *p* < 0.0001).

Thirteen DEGs at D3 vs. D-3 (D3 downregulated: *LALBA*, *CSN2*, *CSN1S2*, *CSN1S1*, *SLC7A5;* D3 upregulated*: MXRA5*, *SLC7A8*, *LBP*, *ANGPTL4*, *LOXL4;* and D3 no change: *CCL28*, *ZO3*, *IGSF3*) were selected to validate RNA-Seq results followed the same direction of expression under qRT-PCR and had comparable log_2_ fold change (Fig. 6A). Expression levels calculated via RNA-Seq were significantly positively correlated to expression levels determined via qRT-PCR (Fig. 6B; R^2^ = 0.96, *p* < 0.0001).

## Discussion

The dry period is characterized by dynamic shifts in mammary gland cellular metabolism, cell turnover, immune signaling, and tissue remodeling. Any perturbation (e.g. exposure to heat stress) of these cellular processes and developmental events could severely reduce the mammary gland’s ability to effectively involute and redevelop, negatively affecting milk production in the next lactation^14,23^. The present study confirms the involvement of metabolic, cell death, and immune-related genes and pathways in the bovine mammary gland during the dry period and reveals others not previously reported. Our findings provide insights into the landscape of the bovine mammary transcriptome undergoing involution when exposed to environmental heat stress, highlighting changes in cell death, branching morphogenesis and cell response to stress.

Cessation of milking induces the recruitment of immune cells and local factors, such as pro-apoptotic signaling factors, and increases mammary pressure. This leads to a dramatic decline in milk synthesis and metabolic processes and protects against inflammation^6,10^. More than 3,000 DEGs between late lactation and early involution and more than 800 DEG during the first week of involution were discovered. After seven days of milk stasis, the mammary gland approaches the end of the active involution phase. Interestingly, there were no DEGs under FDR ≤ 5% during the steady state and redevelopment time-point comparisons (D14 vs. D7 and D25 vs. D14). Possible explanations include failure to capture peak gene expression associated with redevelopment, inability to capture potential changes caused by post-transcriptional modifications, location and heterogeneity of mammary tissue collected, and subtle physiological alterations not captured under our experimental design and statistical analysis.

The most significant pathways downregulated during early involution were related to synthesis and metabolism of lipids, proteins, and carbohydrates. These findings are consistent with previous research where, in general, concentrations of milk-specific constituents decline as lactogenic activity halts in the involuting mammary gland^6,24^. Pathways and terms related to lipid metabolism (e.g. *steroid biosynthesis*, *synthesis and degradation of ketone bodies*, *fatty acid degradation*, *saturated* and *unsaturated fatty acids*) expressed a higher number of downregulated genes, indicating reduced lipid synthesis and metabolism at D3 of involution. Pathways related to biosynthesis, degradation, and transport of amino acid and terms related to milk proteins (e.g. *lactalbumin*, *caseins*, and *lactoglobulins*) had a higher number of downregulated genes at D3 of involution, which is consistent with downregulation of milk protein gene expression and decreased concentrations of milk-specific proteins upon milk stasis^10,25^. Fifteen out of 17 DEGs in the *valine*, *leucine*, *and isoleucine degradation* pathway were also downregulated. Interestingly, some of those genes (e.g. *IVD*, *DBT*, *BCAT2*) are involved in catabolism of the branched-chain amino acids for eventual milk protein synthesis^26,27^. Production of the milk-specific carbohydrate lactose declines rapidly upon milk stasis, accompanied by decreased lactose synthetase activity^11,28^. Six (*UGP2*, *PFKM*, *LALBA*, *GANC*, *HK2*, and *B4GALT1*) of the 11 DEGs in the *galactose metabolism* pathway, related to lactose synthesis and lactose synthetase formation, were downregulated after 3 days of milk stasis. Further, to our knowledge, this study is the first to associate particular genes involved in fatty acid metabolism (e.g. *ACAT2*, *EHHADH*, *ACSM5*, and *PDHA1*), amino acid synthesis (e.g. *BCAT2*, *PDHA1*, and *PPAT*), and carbohydrate metabolism (e.g. *B4GALT2*, *UGP2*, *PFKM*, and *ALDOC*) with the bovine dry period^10,11^.

Cell death is one of the molecular landmarks of involution. Pathways and genes involved in different cell death mechanisms are well described in mouse and bovine models of involution using microarrays and qRT-PCR and are confirmed in the present study utilizing RNA-Seq. However, some discrepancies between animal models are apparent. Accumulation of milk in a mouse model causes local factors to induce apoptosis as soon as 12-hours after milk cessation. For example, LIF phosphorylates the signal transducer STAT3^29^, which downregulates a major survival actor pAkt through induction of PI3-kinase and downregulates IGF1 through upregulation of IGFBP5^9,30,31^. Cell death during involution is not as extensive in the dairy cow, and while many of these factors discussed above were present in our study, their temporal expression pattern was different. In our study, pro-apoptotic factors such as *LIF*, *STAT3*, *IGFBP5*, *CASP9*, *BAX*, and *SOCS3* were all upregulated at D3 of involution, while the survival-signaling factor *AKT1S1* was downregulated. Similarly, elevated levels of apoptosis during the early dry period in Holstein cows are evidenced by upregulation of histological markers and pro-apoptotic genes (e.g. *CASP3* and *IGFBP5*) at D4 of involution^5^. These authors also reported a simultaneous increase in mammary expression of proliferative genes (e.g. *IGF1* and *IGF1-R*) during the early involution (D4) and redevelopment (D36) phases of the dry period. In our study, not *IGF-1* but *IGF1-R*, *IGFBP2* and *IGFBP4* were upregulated in the mammary gland at D3 of involution compared with late lactation. Abruptly drying-off non-pregnant dairy cows at peak lactation increased apoptosis of the mammary gland (D3 to D8 after milk stasis), indicated by increased STAT3 and SOCS3 protein levels and decreased *STAT5* gene expression^12^. However, *IGF1* expression increased and IGFBP5, Akt and Akt-P protein concentrations did not change^12^. Non-pregnant cows gradually dried-off had increased mammary apoptosis from D5 to D14 of involution evidenced by upregulation of *STAT3* and downregulation of *AKT1*, but no changes in IGFBP5 were reported^11^. Additionally, in our study, autophagy-promoting genes (e.g. *ATG9*, *DRAM1*, and *EPG5*) were upregulated in the mammary gland of cows at D3 of involution, demonstrating novel associations between these specific genes and the dry period and corroborating the participation of autophagic cell death in the involuting bovine mammary gland^15,32,33^. Discrepancies between our model and other mouse and bovine models may be attributed to the stage of lactation at dry-off, state of concurrent pregnancy, and reduced extent of MEC turnover during involution. Pro-apoptotic and pro-proliferative molecules may be co-expressed in the mammary gland of pregnant cows that requires both cell death and proliferation during the dry period.

Other molecular landmarks of involution include disruption of cell tight junctions, immune cell signaling, and cytoskeleton and extracellular matrix degradation. Not surprisingly, mammary cell tight junction permeability was impacted by milk stasis^34^. Herein, 15 out of 16 DEGs in the *tight junction* pathway were downregulated during the first week of involution. Immune cell signaling is activated in response to milk stasis to protect against mammary inflammation and remove debris through phagocytosis^35^. In our study, the influx of immune factors was indicated by the upregulation of *bacterial invasion of epithelial cells* and *leukocyte transendothelial migration* pathways and upregulation of immune-related genes (e.g. *LBP*, *TMSB4X*, *ANXA1*, and *STAT3*) after D3 of initiated involution. In addition, genes upregulated in the *lysosome*, *phagosome*, and *peroxisome* pathways (e.g. *SOD*, *LAMP1*, *SORT1*, and *COMP*) indicate clearing of apoptotic cell bodies after D3 of involution. Phagocytosis of apoptotic cells is not pro-inflammatory and acts in a wound-healing manner^36^ by inducing expression of inflammatory factors that were upregulated in our dataset (e.g. *IL34*, *IL27RA*, *IL6R*, *IL10RB*, *IL1R1*). Neutrophil-attracting chemokines (e.g. *CXCL12*, *CXCL13*, and *CXCL17*) were upregulated at D3, in accordance with the pro-inflammatory molecules reported in a mouse model of involution^31^. The observed downregulation of genes involved chemotaxis at D7 of involution is consistent with the reported presence of immune factors in a non-pregnant bovine model at 36 h after milk stasis^12^. Pathways and terms associated with cytoskeleton degradation (e.g. *adherens junction*, *focal adhesion*, *regulation of actin cytoskeleton*, and *actins*) had a greater number of genes upregulated D3 of involution. This was accompanied by upregulation of genes (e.g. *RHOA*) involved in the reorganization of the actin cytoskeleton. As involution progressed to D7, *adherens junction* and *actin cytoskeleton* pathways were downregulated while the stromal matrix metallopeptidase 27 (*MMP27*) was upregulated indicating promotion of extracellular matrix breakdown^35^. Many genes (e.g. *ILK*, *ACTG1*, *LAMP1*, *IL34*, *CXCL12*, *MMP27*, and *RHOA*, *etc*.) involved in these immune-related and cellular structure functions were first associated with the bovine dry period in this study.

Our study revealed novel upstream regulators in the mammary gland during early involution. Two upstream regulators that play central roles in energy metabolism, *PPARGC1A* and *INSIG1*, were downregulated in the mammary gland of dairy cows during early involution, supporting a rapid and coordinated decrease of overall cellular metabolism upon milk stasis. Upstream regulators of lipid synthesis that coordinate downstream target networks were downregulated at D3 of involution, consistent with a previous bovine involution study^11^. The *ACACA* and its lipogenic downstream target genes (*FASN* and *GPAM*) were downregulated. Similarly, *SCD*, a key upstream regulator in oleic acid biosynthesis that interacts with and regulates other upstream regulators (such as *ACACA*, *LPL*, and *SREBF1*) was downregulated. The upstream regulator *ALOX15*, which acts on polyunsaturated fatty acids to generate bioactive lipid mediators that regulate inflammation and immunity, was also downregulated. Three pro-apoptotic factors *IGFBP5*, *PTGES*, and *BACH2* are examples of upstream regulators related to cell death that were upregulated at D3 of involution relative to late lactation in our bovine model. This study is the first to consider the roles of *PTGES* and *BACH2* as cell death mediators in the bovine dry period. As involution progressed to D7, the number of upstream regulators dropped but the majority were upregulated and related to the cell cycle. Specific functions of these factors include mitotic regulation (*NEK2*), chromosome segregation through the spindle checkpoint (*BUB1B*), cyclin dependent kinases (*CKS2*), and regulation of cyclin expression (*FOXM1*). There were three downregulated upstream regulators: *NUPR1*, involved in combating micro-environmental cellular stress, *EFNA1*, modulating developmental events in the vascular system, and *RET*, a cell proliferation and growth signaling molecule. The upstream regulator *NUPR1* is not only a negative regulator of cell cycle but also targets downstream genes that assist stress signaling to fortify cells against perturbations like reactive oxygen species and defective DNA repair, all critical components of immune response and tissue remodeling. Our study revealed novel genes, pathways, upstream regulators and transcription factors that could be targets of future studies to promote more rapid and efficient mammary gland involution, particularly during the early stages.

Interestingly, a non-annotated lncRNA was downregulated in the mammary glands of heat-stressed dry cows compared with cooled cows at D25 relative to dry-off. Long non-coding RNAs are involved in gene regulation through a variety of mechanisms like binding to complementary RNA to affect RNA processing, turnover, or localization or serving as precursors for smaller regulatory RNAs such as microRNAs or piwiRNAs^37^. We identified seven miRNA seed regions within the lncRNA sequence that impact 1,159 downstream target genes, including known markers of involution (e.g. *SOCS3*, *IGF1R*, *IGFR*, *AKTIP*) and upstream regulators that are significantly up- or downregulated during involution or in heat-stressed dry cows (e.g. *PPARGC1A*, *ACACA*, *VEGFA*, *ERBB2*). Recent studies have identified miRNAs differentially expressed between lactating and non-lactating ruminants^38–40^. For example, target genes for miRNAs (e.g. miRNA-148 and miR-145) expressed during the dry period promote cell death by downregulating *STAT5*^40^ and play a role in mammary metabolism by targeting *INSIG1* for lipogenesis. Inhibition of miR-145 in goat MEC led to increased methylation levels of *FASN*, *SCD1*, *PPARG*, and *SREBF1*^41,42^. Thus, the downregulation of the lncRNA by heat stress might affect the regulation of miRNAs, resulting in altered expression of proapoptotic and metabolic genes and key transcription factors involved in mammary gland cell turnover and metabolism. Further investigation is needed to determine how important these miRNAs are in regulating downstream target gene expression in the mammary gland of heat-stressed cows.

Under the less stringent analysis, genes impacted by heat stress play a role in key processes in mammary gland development, such as ductal branching morphogenesis (e.g. *TEKT3*, *WIF1*, *LCA5*, *ACTL8*). Our results support previous reports of aberrant ductal branching of bovine MEC exposed to high temperatures *in vitro*^17^. Genes related to mammary gland function, including immune function (e.g. *FCAMR*, *GP2*, *CRTAC1)*, inflammation (e.g. *ILR20B*, *KLK7*), and cell stress protection (e.g. *DNAJC12*) were upregulated in heat-stressed cows. In an *in vitro* rat model, immune signaling was upregulated to combat heat stress, where activation occurred via extracellular secretions of heat-shock proteins^43^. Similarly, previous literature has reported overexpression of other heat-shock proteins in the mammary gland of rat and bovine models to protect cells against hyperthermia^18,44,45^. The transcriptome profile of dry, heat-stressed cows during early involution is indicative of impaired mammary development, aberrant cellular processes and extended inflammation and immune response.

We recognize that this analysis is exploratory in nature, thus findings warrant further investigation. Possible reasons for the fewer significant differences between heat-stressed and cooled cows include subtle gene expression shifts in our *in vivo* chronic heat stress model at the level of the mammary gland, limited statistical power to capture those differences, variability in location of tissue collection, as our biopsied tissue was collected from alternating quarters at each time point and cell heterogeneity, as cell sorting of mammary epithelial cells was not performed.

## Conclusions

This is the first *in vivo* study to characterize the bovine mammary gland transcriptome during the dry period and under environmental heat stress utilizing RNA-Seq. Regardless of the caveats and limitations of our *in vivo* bovine dry period model and design, our findings reveal novel genes, pathways, and upstream regulators involved in the dynamic process of mammary gland involution and point towards key genes and pathways impacted by dry period heat stress, many of which have never been directly associated with an *in vivo* bovine dry period model previously. From these, upstream regulators including metabolic regulators (e.g. *PPARGC1A* and *INSIG)* and pro-apoptotic regulators (e.g. *IGFBP5*, *PTGES*, and *BACH2*) are ideal candidates for future exploration with the potential to alter expression of key downstream genes. This work serves as the basis for more exhaustive research to investigate these candidate genes and pathways to combat the negative effects of heat stress and promote successful cell turnover and tissue restoration with the goal of improving synthetic capacity for the subsequent lactation.

## Materials and Methods

This study was conducted at the University of Florida Dairy Unit (Hague, FL) over the summer of 2015. The University of Florida Institutional Animal Care and Use Committee approved all treatments and procedures, and all experiments were conducted in accordance with their rules and regulations. Twelve multiparous Holstein cows selected based on mature equivalent milk production and parity were dried off at 46 d before expected calving. Cows were randomly assigned to two treatments for the duration of the dry period: *heat-stressed* (**HT**, n = 6; access to shade in a sand-bedded free-stall pen) or *cooled* (**CL**, n = 6; access to shade, fans and soakers in a separate pen). Fans (J&D Manufacturing, Eau Claire, WI) ran continuously and soakers (Rain Bird Manufacturing, Glendale, CA) were activated when ambient temperature reached 21.1 °C, running for 1.5 min in 6 min intervals. Upon calving, cows were treated identically with access to shade, fans, and soakers. Details of the total mixed ration diet, dry matter intake, rectal temperature and respiration rates during the dry period, and milk production during lactation are reported in Fabris *et al*.^19^.

For all cows, mammary biopsies were collected at day (**D**) −3 (before dry-off during late lactation) and at D 3, 7, 14, and 25 relative to dry-off (which was considered D0) based on the method described by Farr *et al*.^46^ with slight modifications^14^. Time points for mammary biopsy collection were chosen to capture the three phases of the dry period: D-3 represents late lactation, D3 and D7 represents active involution, D14 represents the steady-state phase, and D25 captures the beginning of the redevelopment phase. Mammary tissue biopsies were washed in sterile saline, trimmed of visible fat, placed in RNAlater (ThermoFisher, Invitrogen, Grand Island, NY), and stored at −80 °C until RNA isolation. Total RNA was extracted using the RNeasy Mini Kit (catalog #74104, Qiagen, Valencia, CA) according to the manufacturer’s instructions. RNA concentration was determined on Qubit 2.0 Fluorometer (ThermoFisher, Invitrogen, Grand Island, NY), and RNA quality was assessed using the Agilent 2100 Bioanalyzer (Agilent Technologies, Inc.). Total RNA with 28 S/18 S >1 and RNA integrity number ≥7 were used for library construction.

RNA-Seq library was constructed using NEBNext Ultra RNA Library Prep Kit for Illumina (New England Biolabs, USA) following manufacturer’s recommendations. Briefly, 500 ng of total RNA was used for mRNA isolation using NEBNext Poly(A) mRNA Magnetic Isolation module (catalog #E7490) then followed by RNA library construction with NEBNext Ultra RNA Library Prep Kit for Illumina (catalog #E7530) according to the manufacturer’s user guide. Sixty barcoded libraries (n = 12 cows at 5 different time points D-3, 3, 7, 14, 25) were sized on the Bioanalyzer, quantitated by QUBIT and quantitative PCR using the KAPA library quantification kit (Kapa Biosystems, catalog #KK4824). Finally, the 60 individual libraries were pooled equimolarly and sequenced by Illumina NextSeq. 500 for 5 runs (Illumina Inc., CA) which generated 150 base-pair single-ended reads.

The quality of the sequencing reads was evaluated using FastQC software, and if necessary, sequencing reads were trimmed using the software Trim Galore (v0.4.1). Sequence reads were mapped to the bovine reference genome (bosTau7) using the software package Tophat^47,48^ (v2.0.13). Two rounds of alignment were performed to maximize sensitivity to splice junction discovery, allowing for full utilization of novel splice junctions. Novel splice junctions were first determined in each sample individually, then combined with the known ENSEMBL annotated splice junctions and entered in Tophat for a second alignment^49,50^. Read alignments were discarded if they had greater than two mismatches or were equally mapped to more than 40 genomic locations. The subsequent alignments were used to reconstruct transcript models using the software package Cufflinks^51^ (v2.2.1). The Cuffmerge tool was used to merge each assembly to the bovine annotation file, combining novel transcripts with known annotated transcripts to maximize quality of the final assembly. The number of reads that mapped to each gene in each sample was calculated using the tool *htseq-count*^52^.

Differentially expressed genes were detected using the R package edgeR (v.3.4.2)^53^. This package combines the use of the trimmed mean of M-values as the normalization method of the count data, an empirical Bayes approach for estimating tagwise negative binomial dispersion values, and finally, generalized linear models and quasi-likelihood F-test for detecting differentially expressed genes for the main effects of treatment, time, and treatment by time interaction. The following comparisons over time were made: D3 vs. D-3, D7 vs. D3, D14 vs. D7, and D25 vs. D14 to highlight differences in gene expression as the cow transitions between dry period phases, focusing on the active involution phase. Additionally, HT vs. CL treatments were compared for each time point independently. Because there was no significance in the interaction, the main effects of treatment and time were analyzed separately.

Genes that were differentially expressed over time or between treatments were analyzed using Fisher’s exact test to determine significant enrichment of Gene Set Enrichment Analysis Gene Ontology (**GO**) Kyoto Encyclopedia of Genes and Genomes (**KEGG**) pathways and Medical Subject Headings (**MeSH**) terms^54,55^. For all comparisons, genes that had an ENSEMBL annotation and a false-discovery rate (**FDR**) ≤ 5% were tested against the background set containing all expressed genes with ENSEMBL annotation. The GO, KEGG and MeSH enrichment analyses were performed in the R environment using *goseq*.^56^ and *meshr*^57^ packages respectively. Functional categories with a nominal *p* < 0.05 were considered significantly enriched by DEGs.

Additionally, DEGs were explored using Ingenuity Pathway Analysis (**IPA**, QIAGEN Inc., https://www.qiagenbioinformatics.com/products/ingenuitypathway-analysis)^58^ to determine upstream regulators. For each comparison, lists of DEGs with ENSEMBL annotation were uploaded into IPA and compared to the background annotated bovine genome (24,616 unique ENSEMBL IDs). Both up- and downregulated genes were analyzed together. The IPA feature *Upstream Analysis* was used to determine significant upstream regulators within our dataset. IPA broadly describes upstream regulators as any molecule that can affect the expression of other molecules. The impact of upstream regulators was calculated using overlapping p-value to identify regulators that explained observed gene expression changes and activation z score to estimate the activation state of predicted regulators. From this list of upstream regulators, IPA generates a molecular network of upstream regulators, downstream target genes, and biological functions that are impacted by expression changes in these molecules.

Thirteen DEGs were chosen for validation of RNA-Seq results - five DEGs downregulated at D3 (*α-lactalbumin*, *LALBA; β-casein*, *CSN2; casein-αS1; CSN1S1; casein-αS2*, *CSN1S2; solute carrier family 7 member 5*, *SLC7A5*), five DEGs upregulated genes at D3 (*matrix-remodeling-associated protein 5*, *MXRA5; lipopolysaccharide binding protein*, *LBP; lysyl oxidase like 4*, *LOXL4; angiopoietin like 4*, *ANGPTL4; solute carrier family 7 member 8*, *SLC7A8*), and three genes with similar expression at D3 and D-3 (immunoglobulin superfamily, member 3, *IGSF3*; C-C motif chemokine ligand 28, *CCL28*; tight junction protein 3, *ZO3*). Validation was performed using quantitative real-time PCR (**qRT-PCR**) conducted with the CFX96 Touch Real-Time PCR Detection System (Bio-Rad). The same RNA samples were used for RNA-Seq and for technical validation by qRT-PCR. A detailed description of primer design and sequences can be found in Supplementary Methods.

### Accession codes

Sequencing data can be accessed through NCBI GEO with accession number GSE108840. Please access the following link: https://www.ncbi.nlm.nih.gov/geo/query/acc.cgi?acc=GSE108840.

## Electronic supplementary material

Supplementary Methods and Figures

Supplementary Tables
