# Supplementary material for: RNA-Seq reveals novel genes and pathways involved in bovine mammary involution during the dry period and under environmental heat stress
Source: Sci Rep. 2018 Jul 23;8:11096. doi: 10.1038/s41598-018-29420-8 (PMC6056563; doi:10.1038/s41598-018-29420-8)
Supplement: Supplementary file 1 — Supplementary Methods and Figures [file 41598_2018_29420_MOESM1_ESM.pdf]

Supplementary Methods

RNA-Seq reveals novel genes and pathways involved in bovine mammary involution during the dry period and under environmental heat stress. Bethany Dado-Senn, Amy L. Skibiel, Thiago F. Fabris, Y. Zhang, Geoffrey E. Dahl, Francisco Peñagaricano, Jimena Laporta.

RT-PCR methods used for confirmation of RNA-Sequencing. Ten genes in the mammary gland of dairy cows at D3 and D-3 relative to dry off (D0) were used for validation. A total of 1 µg RNA from each sample was used to synthesize cDNA using the iScript cDNA synthesis kit (Bio-Rad Laboratories, CA) and diluted 1:5 in dH_2O. Reaction mixtures were performed as previously described[1] and cycling conditions were as follows: 1 cycle for 3 min at 95°C then 50 cycles of 10 s at 95°C and 30 s at 60°C followed by melt curve measurement from 65°C to 95°C in 0.5° increments for 5 s. Positive and negative (non-template control, **NTC**) controls were added to each PCR plate. Each sample was assessed in duplicate and the %CV between the duplicates was < 2%. Primers sequences for the validated genes were obtained from the literature or specifically designed to span exon-exon junctions to minimize the potential of amplifying genomic DNA using Primer3 software[2,3] (see table). The geometric mean between two housekeeping genes (ribosomal protein S9, ***RPS9*** and ubiquitously expressed prefoldin-like chaperone, ***UXT***) was used to calculate the relative gene expression using the method $2^{-\Delta\Delta Ct}$ with D3 relative to dry off as the reference group[4]. Specificity of amplification for each primer pair was evaluated by plotting the dissociation-characteristics of double-stranded DNA. The figure in this material shows the melt curves for the ten different amplicons. The single peak following melt curve analysis observed for all amplicons is indicative of a pure, single amplicon.

Table. Primer sequences for validated and housekeeping genes.

Gene Name and Symbol	Accession Number	5' ->3'	Primer Sequence	Source
α-lactalbumin (*LALBA*)	BC102173.1	F	AAAGACTTGAAGGGCTACGGA	5
		R	AGATGTTGCTTGAGTGAGGGTT	
β-casein (*CSN2*)	BC111172.1	F	AGTGAGGAACAGCAGCAAACAG	5
		R	AGCAGAGGCAGAGGAAGGTG	
casein-αS1 (*CSN1S1)*	BC109618.1	F	TACCTGTCTTGTGGCTGTTGC	5
		R	CCTTTTGAATGTGCTTCTGCTC	
casein-αS2 (*CSN1S2)*	BC114773.1	F	GCCTGGACTACTTGTCTTCCTTTTA	5
		R	TCCTCTTCATTTGCGTTCCTTAC	
solute carrier family 7 member 5 (*SLC7A5*)	BC126651	F	GGGTGACGTAGCCAATCTGG	6
		R	ATCCCCCATAGGCAAAGAGG	
matrix-remodeling-associated protein 5 (*MXRA5)*	XP_001254410.3	F	CGCTGGGATCTCTCCACAT	2, 3
		R	GAGCTCCAGCTTCGTCAGTC	
lipopolysaccharide binding protein (*LBP)*	NM_001038674	F	TGGAGGTGCACATATCAGGA	2, 3
		R	CTTGCTCTCCAAGACCCTTC	
lysyl oxidase like 4 *(LOXL4)*	NM_174384	F	CCAGCTTCTGCCTAGAGGAC	2, 3
		R	TAGGTATCCCAGCAGCCAAC	
angiopoietin like 4 (*ANGPTL4)*	NM_001046043	F	GAAGAGGCTGCCCAAGATG	2, 3
		R	CCCTCTTCAAACAGCTCCTG	
solute carrier family 7 member 8 (*SLC7A8)*	NM_001192889.2	F	TCAAGGCTCCTTTGCCTATG	2, 3
		R	CAAATGTGACCAGTGGGATG	
ubiquitously expressed prefoldin-like chaperone (*UXT)*	XM_004022128.3	F	TGTGGCCCTTGGATATGGTT	7
		R	GGTTGTCGCTGAGCTCTGTG	
C-C motif chemokine ligand 28 (*CCL28*)	NM_001101163.1	F	ACTTGGCTGCTGTCATCCTT	2
		R	CCTCTTTCTTGGCTGCTTGT	
Immunoglobulin superfamily, member 3 (*IGSF3*)	NM_001192700.2	F	CAGCACTCTCGTCTCCATCA	2
		R	ATGGGGTTTGATGATGCACT	
Tight junction protein 3 (*ZO3*)	NM_001045874	F	GATACCCCGTTCCAGAACCT	2
		R	TGTAGAAGGAGTCGCCCAAG	
ribosomal protein S9 (*RPS9)*	NM_001101152	F	GGAGACCCTTCGAGAAGTCC	1
		R	CTTTCTCATCCAGCGTCAGC	

Figure. Melting curves for the 13 DEGs identified by RNA sequencing validated by RT-PCR.

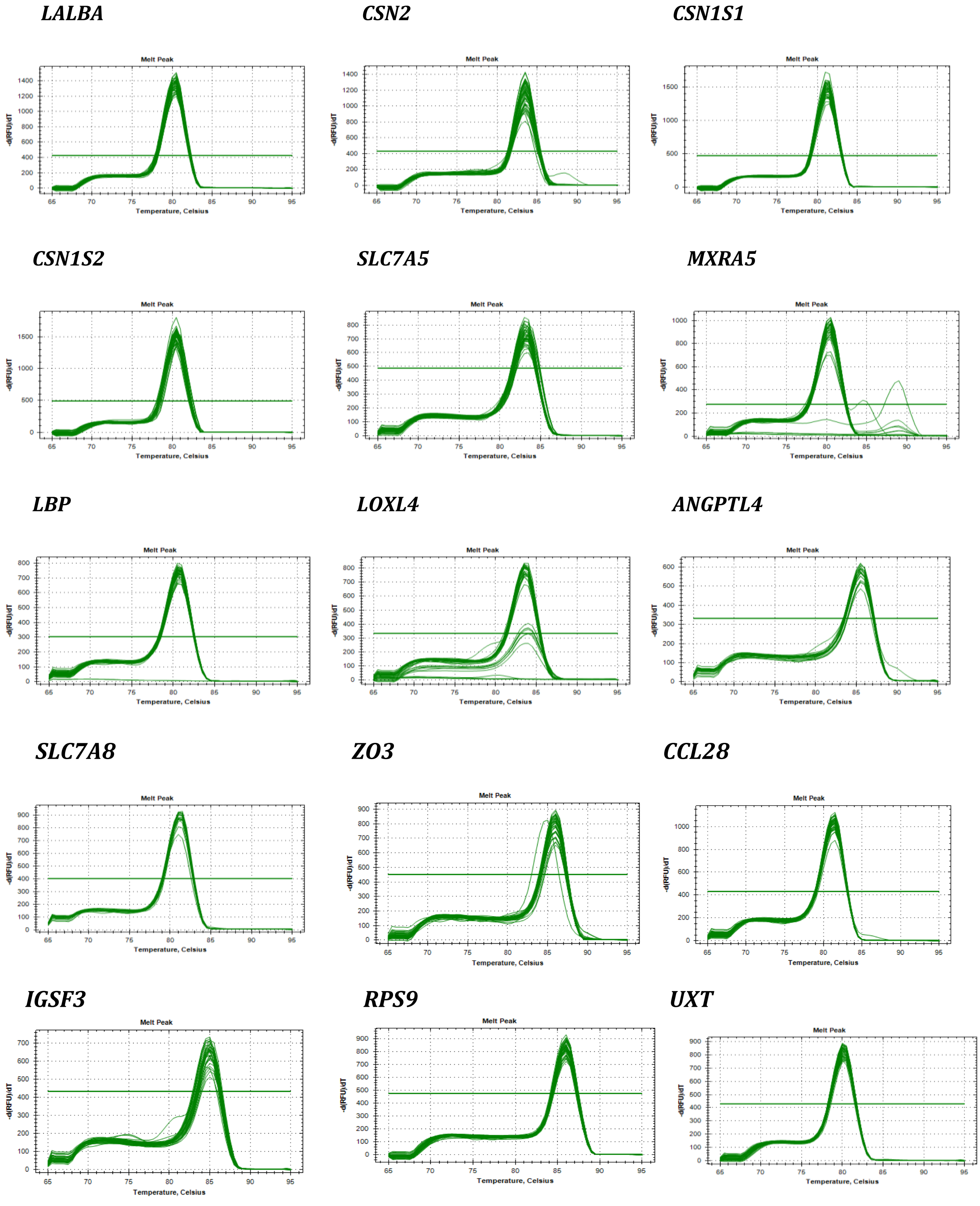

References

1. Laporta, J. L., Keil, K. P., Vezina, C. M., & Hernandez, L. L. Peripheral serotonin regulates maternal calcium trafficking in mammary epithelial cells during lactation in mice. *PlosOne* **9**, e110190 (2014).
2. Untergasser *et al.* Primer3 – new capabilities and interfaces. *Nucleic Acids Research* **40**, e115 (2012).
3. Koressaar, T. & Remm, M. Enhancements and modifications of primer design program Primer3. *Bioinformatics* **23**, 1289-1291 (2007).
4. Livak, K. J. & Schmittgen, T. D. Analysis of relative gene expression data using Real-Time Quantitative PCR and the $2^{-\Delta\Delta Ct}$ method. *Methods* **25**,402-408 (2001).
5. Nan, X. *et al*. Ratio of lysine to methionine alters expression of genes involved in milk protein transcription and translation and mTOR phosphorylation in bovine mammary cells. *Physiological Genomics* **46**, 268-275 (2014).
6. Bionaz, M. & Loor, J. J. Gene networks driving bovine mammary protein synthesis during the lactation cycle. *Bioinformatics and Biology Insights* **5**, 83 (2011).
7. Bionaz, M. & Loor, J. J. Identification of reference genes for quantitative real-time PCR in the bovine mammary gland during the lactation cycle. *Physiological Genomics* **29**, 312-319 (2007).

Supplementary Figures

RNA-Seq reveals novel genes and pathways involved in bovine mammary involution during the dry period and under environmental heat stress. Bethany Dado-Senn, Amy L. Skibiel, Thiago F. Fabris, Y. Zhang, Geoffrey E. Dahl, Francisco Peñagaricano, Jimena Laporta.

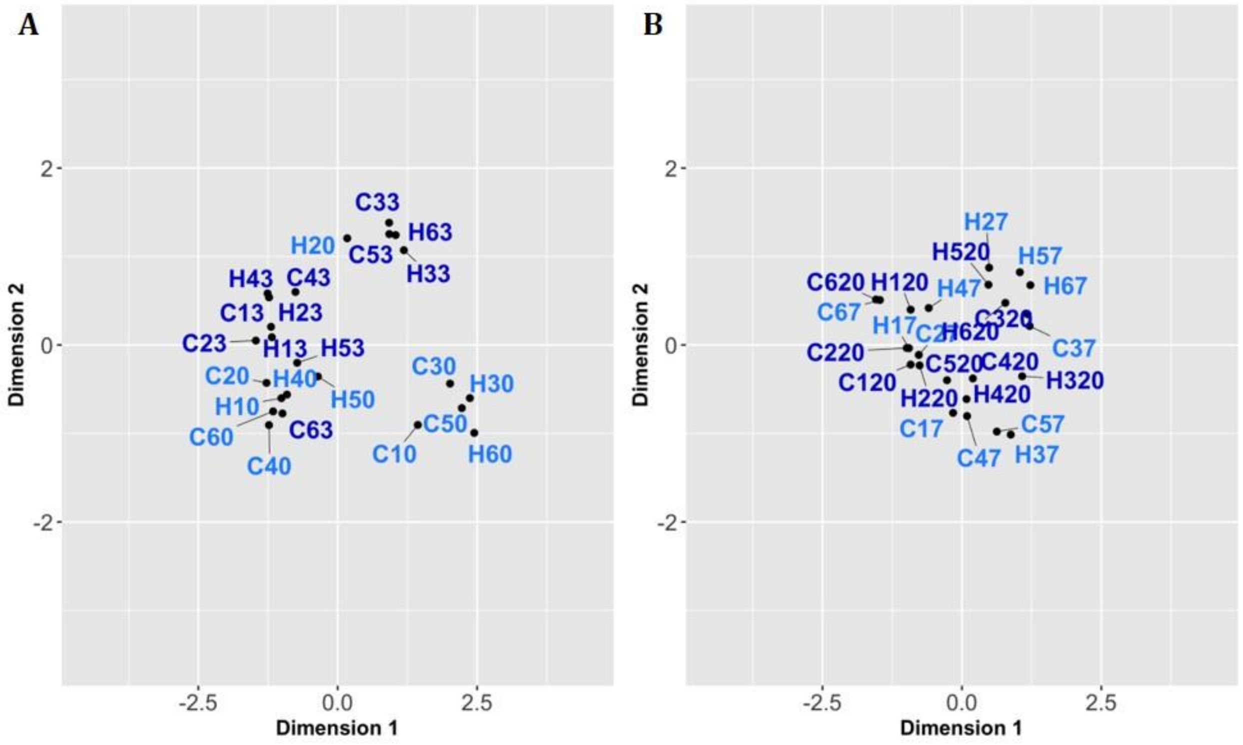

Supplementary Figure S1. Multidimensional scaling (MDS) plots showing the relative similarities of the samples under study. MDS plots of samples from the bovine mammary gland at (**A**) D3 vs. D-3 (n=12, early involution vs. late lactation) and (**B**) D25 vs. D7 (n=12, redevelopment vs. involution). D0 indicates dry-off (~46 d relative to expected calving). C and H indicate cooled or heat-stressed samples, respectively.

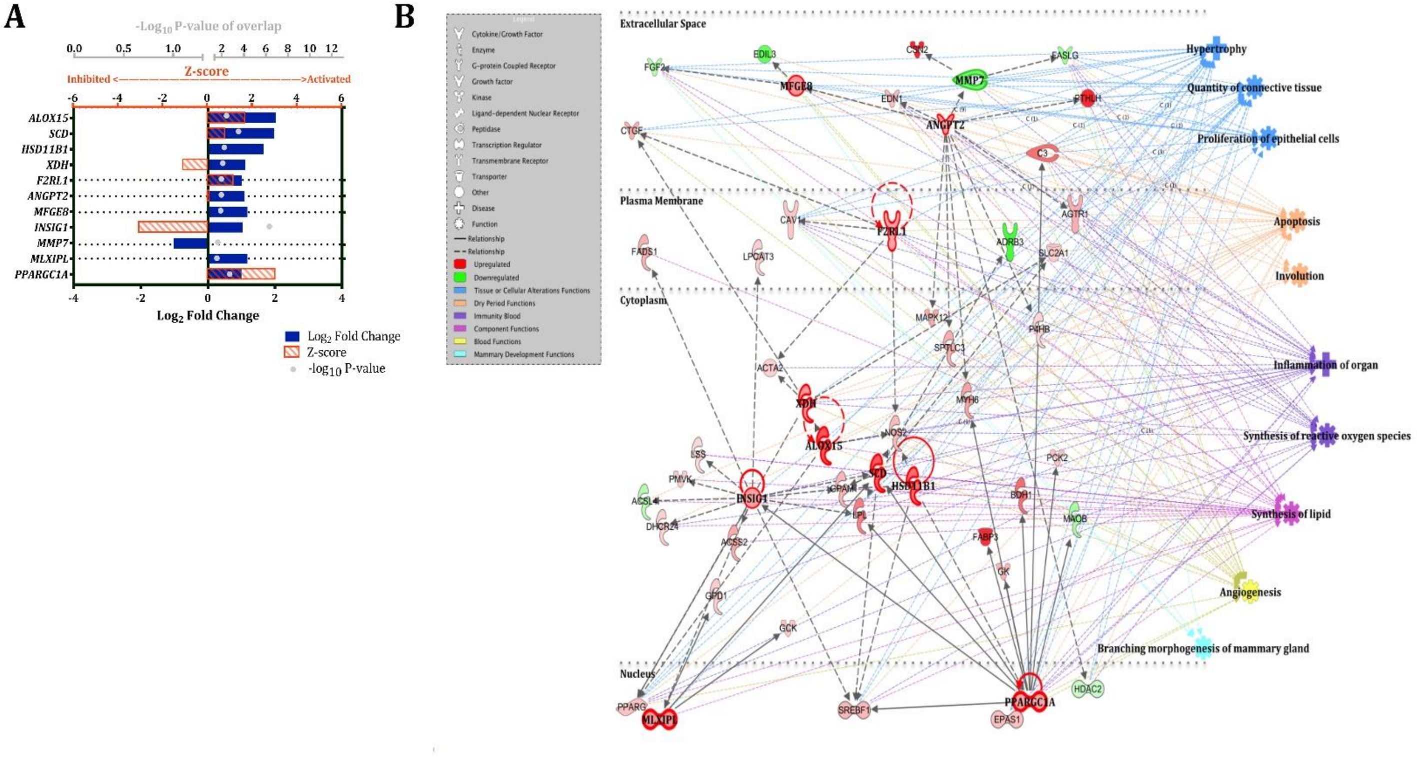

Supplementary Figure S2. Ingenuity Pathway Analysis (IPA) upstream regulators and summary network in bovine mammary tissue between heat-stressed (HT) and cooled (CL) dairy cattle during the dry period. Significant upstream regulators and network in the bovine mammary gland in HT vs. CL cows (relative to HT) at D7 relative to dry-off (D0). The DEG significance was set at a nominal $p \leq 0.005$ with $\log_2$ fold change $\geq|0.05|$ and upstream regulator significance of enrichment at $p \leq 0.05$ with $\log_2$ fold change $\geq|1.0|$. (A) Upstream regulators are grouped by functional categories with $\log_2$ fold change (equivalent to expression log ratio) in blue bars, Z-score (activated: >2, inhibited: <-2) in orange bars, and significance of enrichment (–log10 P-value) in gray dots. (B) The summary network depicts the interactions between upstream regulators, downstream genes, and physiological functions impacted by heat stress. Red and green molecules indicate upregulated and downregulated genes in HT at D7, respectively, relative to CL at D7. Figure legend displays molecules and function symbol types and colors. The functional networks were generated through the use of IPA (QIAGEN Inc., https://www.qiagenbio-informatics.com/products/ingenuity-pathway-analysis).
